# Acute Pediatric Myelin Oligodendrocyte Glycoprotein (MOG) Antibody-Associated Rhombencephalitis With Multifocal Brain Involvement: A Case Report and Discussion

**DOI:** 10.7759/cureus.104166

**Published:** 2026-02-24

**Authors:** Ruilin Wang, Jessica N Smock, Anusri Pakhare, Anna Debonaventura, Kristie Rivers

**Affiliations:** 1 Osteopathic Medicine, Nova Southeastern University Dr. Kiran C. Patel College of Osteopathic Medicine, Fort Lauderdale, USA; 2 Pediatrics, Broward Health, Fort Lauderdale, USA; 3 Pediatrics, Broward Health Medical Center, Fort Lauderdale, USA

**Keywords:** autoimmune neuroinflammation, myelin oligodendrocyte glycoprotein antibody-associated disease (mogad), pediatric demyelinating disease, pediatric neuroimmunology, rhombencephalitis

## Abstract

Rhombencephalitis is a rare inflammatory condition involving the brainstem, with both infectious and autoimmune causes. While infections account for many pediatric cases, autoimmune etiologies such as myelin oligodendrocyte glycoprotein antibody-associated disease (MOGAD) have gained wider recognition.

We present a six-year-old male with no significant past medical history who presented with acute-onset ataxia, dysarthria, olfactory hallucinations, and headache with associated intermittent fever. Cerebrospinal fluid (CSF) analysis revealed leukocytosis with increased lymphocytes and monocytes, but negative infectious studies. Two days after the patient initially presented, brain magnetic resonance imaging (MRI) revealed findings suggestive of autoimmune rhombencephalitis. After administration of intravenous (IV) methylprednisolone and IV immunoglobulin G (IgG), the patient's condition drastically improved. Due to laboratory processing timelines, the myelin oligodendrocyte glycoprotein antibody (MOG-IgG) resulted positive 10 days after the patient’s initial presentation and five days after the patient had clinically returned to baseline and been discharged.

MOGAD in children is known to have a variety of clinical presentations, and delayed antibody test results should not postpone initiation of acute immunosuppressive therapy. Acute treatment should be initiated based on clinical and radiological suspicion to prevent persistent neurological deficits and risk of long-term disability.

## Introduction

Rhombencephalitis refers to the inflammatory involvement of the brainstem and cerebellar structures and, although uncommon, can lead to significant neurologic compromise [1]. There are numerous etiologies of this inflammation, including autoimmune diseases, infections, and paraneoplastic syndromes [2]. This pathology is particularly rare in children, and when it does occur in pediatric populations, it is most often attributed to infectious causes, with *Listeria monocytogenes* representing the most commonly reported cause, followed by enterovirus A71 and, less frequently, herpes simplex virus types 1 and 2 [2].

Less commonly, rhombencephalitis is caused by autoimmune inflammation. Numerous different autoimmune processes can be involved, but some of the most well-recognized etiologies include multiple sclerosis, myelin oligodendrocyte glycoprotein antibody-associated disease (MOGAD), neuromyelitis optica spectrum disorder, acute hemorrhagic leukoencephalitis, autoimmune glial fibrillary acidic protein astrocytopathy, chronic lymphocytic inflammation with pontine perivascular enhancement responsive to steroids, Bickerstaff brainstem encephalitis, and Behçet disease [3].

Each autoimmune process presents with distinct clinical features, making timely recognition critical. Identifying whether an autoimmune etiology is present and determining the specific disorder is essential, since many neurologic deficits can be reversed with prompt and appropriate treatment [3].

MOGAD is characterized by acute inflammatory attacks in which activated T cells and anti-MOG antibodies cross the blood-brain barrier and induce demyelination within the central nervous system [4]. Myelin oligodendrocyte glycoprotein (MOG) is a critical component of oligodendrocyte surface membranes, where it contributes to the formation, maintenance, and turnover of myelin sheaths. MOG is a distinct member of the immunoglobulin superfamily, containing an extracellular immunoglobulin variable domain, a hydrophobic transmembrane region, a short cytoplasmic loop, a second hydrophobic membrane-spanning domain, and a cytoplasmic tail [5]. This structural configuration, combined with MOG’s location on the outermost surface of the myelin sheath, renders it readily accessible to immune-mediated attack by antibodies and T cells [5].

Elevated titers of autoantibodies directed against MOG have been identified across a range of demyelinating conditions, including optic neuritis, transverse myelitis, acute disseminated encephalomyelitis (ADEM), and cerebral cortical encephalitis [4]. These disorders are now acknowledged as part of a unified spectrum of anti-MOG antibody-associated diseases, collectively classified as MOGAD [4]. These MOGAD disorders need to be distinguished from other demyelinating diseases, mainly multiple sclerosis and aquaporin-4-seropositive neuromyelitis optica spectrum disorder [6].

The diagnostic criteria for MOGAD require fulfillment of three categories. The first requirement is the presence of a core clinical demyelinating event. More specifically, the patient must have experienced one or more of the five recognized MOGAD-associated syndromes, which are optic neuritis, transverse myelitis, ADEM, brainstem/cerebellar syndromes, or cerebral cortical encephalitis [6]. The second requirement is a clear positive myelin oligodendrocyte glycoprotein immunoglobulin G (MOG-IgG) defined by the individual assay cutoff or as a fixed cell based assay with a titer of ≥1:100. If this titer is low positive defined by individual assay cutoff or by a titer of ≥1:10 and <1:100, positive without a reported titer, or negative but with a clear positive MOG-IgG from the CSF then additional supporting features are required to make the diagnosis [6]. These features include aquaporin 4-IgG seronegativity and at least one supporting clinical or MRI feature. Supporting clinical or MRI features are grouped into three categories, corresponding to findings that support a diagnosis of optic neuritis, myelitis, or a brain, brainstem, or cerebral syndrome. The third and final requirement for diagnosis of MOGAD is the exclusion of another better diagnosis, including multiple sclerosis [6].

## Case presentation

A previously healthy six-year-old male with no significant past medical history presented to the emergency room with slurred speech, progressive gait instability, intermittent blurred vision, olfactory hallucinations, and horizontal nystagmus. On the day of admission, he reported a severe bilateral headache that had previously reached 10 out of 10 in intensity and resolved spontaneously. He denied numbness, tingling, vision changes, head trauma, nausea, or vomiting. The patient was hemodynamically stable, non-febrile, and saturating well on room air. He was born full term with no complications and up to date on vaccinations. Family history was notable for maternal migraines and spondylolisthesis, as well as a grandparent with systemic lupus erythematosus. Initial evaluation revealed an unremarkable complete blood count, basic metabolic panel, toxicology screen, non-contrast head computed tomography (CT), electrocardiogram, and chest radiograph. The respiratory viral panel was negative, and the erythrocyte sedimentation rate was mildly elevated at 19 mm/hr (reference range: <15 mm/hr).

During the first two days of hospitalization, the patient developed two febrile episodes with maximum temperatures of 38.6°C and 38.3°C, both of which were resolved with a single dose of oral acetaminophen at 15 mg/kg. Physical examination was notable for dysarthria, dysmetria, dysdiadochokinesia, and an unsteady gait, with the remainder of the neurologic examination being unremarkable. Lumbar puncture was performed on day two and showed a cerebrospinal fluid (CSF) white blood cell count of 58/mm^3^ (reference range: < 7/mm^3^) with 66% lymphocytes (reference range: 25%-33%) and 30% monocytes (reference range: 3%-7%), consistent with a non-bacterial inflammatory process (Table 1).

**Table 1:** Relevant laboratory values obtained during hospitalization, including pediatric reference ranges and the day of collection relative to admission. ESR: erythrocyte sedimentation rate; CSF: cerebrospinal fluid; WBC: white blood cells.

Parameter	Value	Units	Reference range (Pediatric)	Day of admission	Interpretation
ESR	19	mm/hr	<15	Day 1	Mildly elevated
CSF, WBC	58	cells/mm^3^	0-10	Day 2	Elevated
CSF, lymphocytes	66	%	20-60	Day 2	Mildly elevated
CSF, monocytes	30	%	10-20	Day 2	Mildly elevated
Temperature, axillary	38.6	C	<38	Day 1	Fever
Temperature, axillary	38.3	C	<38	Day 2	Fever

The infectious workup included CSF bacterial culture, a multiplex viral CSF PCR panel (herpes simplex virus, enterovirus, Listeria, etc.), and blood cultures, all of which were negative. Cultures were read at 48 hours and 72 hours, both times negative for microbial growth, making bacterial rhombencephalitis unlikely. Liver enzymes, bilirubin, and creatine kinase were within normal limits. Blood culture and throat culture were negative at 48 hours and 72 hours. CT angiography of the head and neck demonstrated normal intracranial and extracranial vasculature without evidence of stenosis, aneurysm, or occlusion.

**Figure 1:**
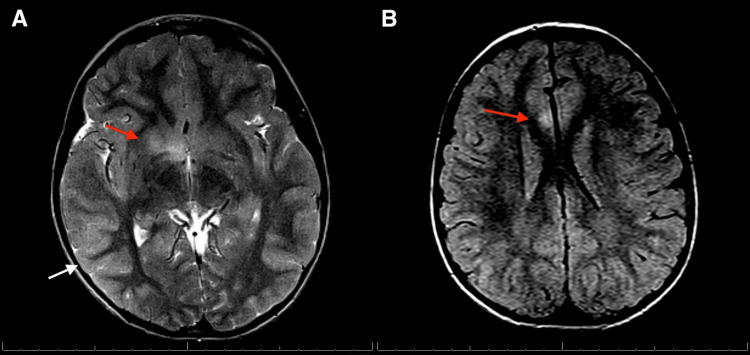
Axial T2-weighted and FLAIR MRI of the brain demonstrates an ill-defined, patchy area of T2/FLAIR hyperintensity involving the inferior margin of the right basal ganglia, located at the medial margin of the right anterior commissure (A, red arrow). Additionally, there is subtle cortical FLAIR hyperintensity involving the bilateral posterior temporal lobes (A, white arrow), extending to the temporo-occipital junctions. A linear strip of FLAIR hyperintensity is also noted along the right cingulate gyrus (B, red arrow). FLAIR: fluid-attenuated inversion recovery.

**Figure 2:**
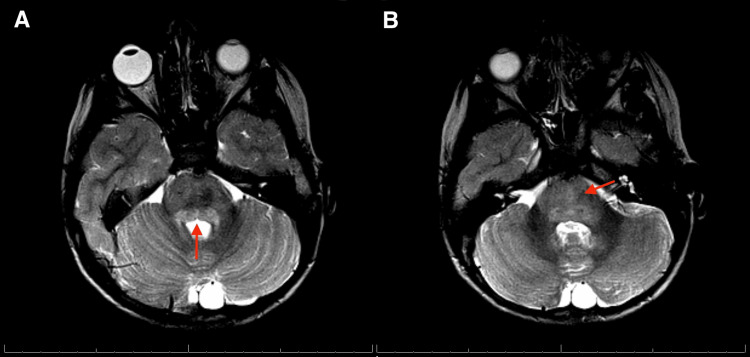
Axial T2-weighted MRI of the brainstem demonstrates diffuse T2 hyperintensity throughout the pons extending from the pontomedullary junction (A, red arrow) to the mesencephalic junction with involvement of the dorsal pons, including the periaqueductal gray (B, red arrow). The image findings are consistent with an inflammatory process involving the brainstem.

**Figure 3:**
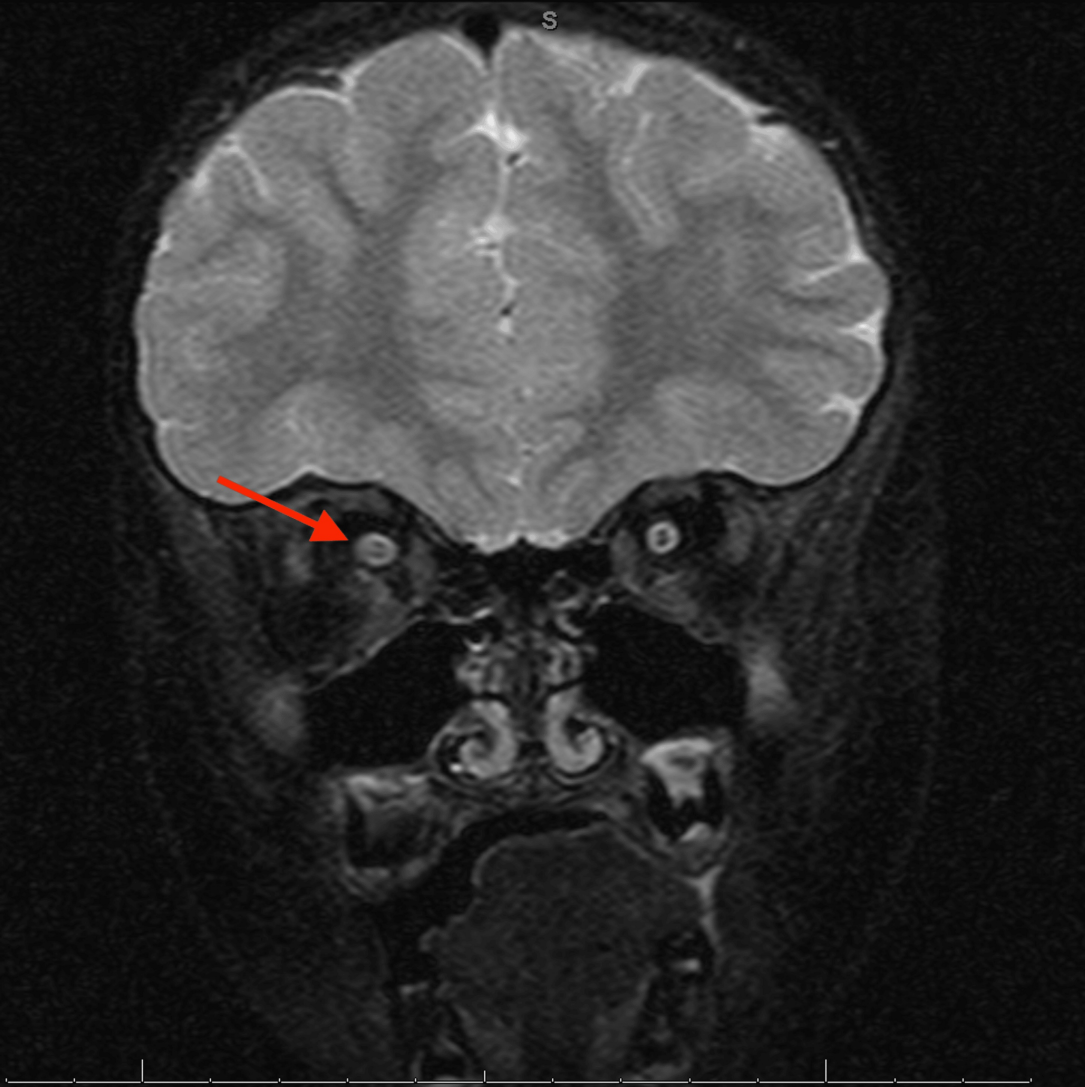
Coronal T2-weighted MRI of the orbits demonstrates questionable increased T2 signal involving the intraorbital segment of the right optic nerve (red arrow), without definite enlargement or abnormal enhancement.

Magnetic resonance imaging (MRI) of the brain with and without contrast showed poorly demarcated T2 and fluid-attenuated inversion recovery (FLAIR) hyperintensities in the pons, right basal ganglia, temporal lobe, temporal occipital junctions, and the right cingulate gyrus, raising suspicion for demyelination disorders and rhombencephalitis (Figures 1, 2). MRI of the orbit showed increased T2 signal of the right optic nerve, concerning for optic neuritis (Figure 3). MRI of the cervical, thoracic, and lumbar spine showed no evidence of myelitis. The ophthalmology service reported visual acuity of 20/50 in the right eye and visual acuity of 20/20 in the left eye.

Due to the concern for autoimmune involvement, serum and CSF antibody panels were ordered, which included myelin oligodendrocyte glycoprotein antibody (anti-MOG) immunoglobulin G (IgG) and immunoglobulin M (IgM), aquaporin-4 IgG (AQP4-IgG), antinuclear antibody (ANA), anti-Smith (anti-Sm) antibodies, anti-double-stranded DNA (anti-dsDNA) antibodies, as well as numerous anti-neuronal autoantibodies (Table 2). Results remained pending throughout the hospital stay due to extended laboratory processing times, and it was later determined that the patient had a positive serum anti-MOG IgG.

**Table 2:** Serum and cerebrospinal fluid (CSF) autoimmune and paraneoplastic antibody testing was performed using immunofluorescence assays (IFA) and fixed cell-based assays (CBA), as appropriate.

Antibody	Serum result	CSF result
Myelin oligodendrocyte glycoprotein (MOG) antibody	Positive	Negative
ANNA-1 (Hu)	Negative	Negative
ANNA-2 (Ri)	Negative	Negative
ANNA-3	Negative	Negative
PCA-1 (Yo)	Negative	Negative
PCA-2	Negative	Negative
PCA-Tr (DNER)	Negative	Negative
AGNA/SOX1	Negative	Negative
Amphiphysin	Negative	Negative
CRMP5/CV2	Negative	Negative
GAD65	Negative	Negative
Ma2/Ta	Negative	Negative
Myelin antibody	Negative	Negative
Aquaporin-4 (NMO) antibody (IFA)	Negative	Negative
Aquaporin-4 antibody (CBA)	Negative	Negative
NMDA receptor (NMDAR1) antibody	Negative	Negative
AMPA receptor 1 antibody	Negative	Negative
AMPA receptor 2 antibody	Negative	Negative
GABA-B receptor antibody	Negative	Negative
LGI1 antibody	Negative	Negative
CASPR2 antibody	Negative	Negative
DPPX receptor antibody	Negative	Negative
Voltage-gated potassium channel (VGKC) antibody	Negative	Negative
Acetylcholine receptor ganglionic (α3) antibody	Negative	Not tested
Voltage-gated calcium channel (P/Q-type) antibody	Negative	Not tested
Voltage-gated calcium channel (N-type) antibody	Negative	Not tested

The patient remained hemodynamically stable for the rest of the hospital stay, and the neurology service was consulted. Autoimmune rhombencephalitis was suspected due to the MRI results, so the patient was given intravenous immunoglobulin (IVIG) 1 g/kg for two days, and IV methylprednisolone 30 mg/kg for four days starting from day two of hospital admission. Caregivers reported rapid improvement of the patient’s ataxia, slurred speech, and activity level within six hours of IV methylprednisolone and IVIG administration. Repeated neurological exams showed no recurrence of ataxia, dysarthria, dysmetria, or dysdiadochokinesia, and the patient was subsequently discharged with neurology outpatient follow-up.

## Discussion

MOGAD can present with a wide range of clinical manifestations, including optic neuritis, focal neurological deficits, ADEM, cerebral cortical encephalitis, and rhombencephalitis [6]. The diagnosis is established by the presence of a demyelinating event, detection of MOG-IgG in the serum, or characteristic MRI features, such as optic neuritis, myelitis, or multiple ill-defined T2 hyperintense lesions involving the cerebral or brainstem regions, and exclusion of alternative diagnoses, including multiple sclerosis (MS) [6].

In this case, the patient’s gait instability, slurred speech, blurred vision, negative infectious studies, and multifocal MRI lesions raised concern for a pediatric acquired demyelinating disorder. The initial differential diagnosis included MOGAD, neuromyelitis optica spectrum disorder (NMOSD), and MS based on clinical presentation and imaging findings [7]. MS was considered less likely given the absence of well-demarcated T2 lesions, T1 hypointensities, and typical periventricular, corpus callosum, juxtacortical, or cortical involvement [6]. NMOSD was also initially suspected due to overlapping MRI features; however, this diagnosis was ruled out by negative aquaporin-4 IgG testing in both serum and cerebrospinal fluid. Imaging from our patient demonstrated pontine involvement, and his brainstem and cerebellar symptoms (ataxia, dysarthria, and diplopia) supported a rhombencephalitis-predominant presentation. Fever initially raised concern for infection, but the negative evaluation and rapid steroid and IVIG response favored an immune-mediated demyelinating process.

An important clinical decision in this case was the initiation of high-dose intravenous corticosteroids and intravenous immunoglobulin prior to confirmation of antibody results. This approach was supported by several factors. First, while olfactory hallucinations are a rare associated symptom, the MRI findings of poorly demarcated T2/FLAIR hyperintensities involving the brainstem, basal ganglia, and cortical regions were consistent with known radiographic patterns of MOGAD. Second, multiple studies have demonstrated that early intravenous steroid therapy in the acute setting improves speed of recovery and neurologic outcomes in MOG-IgG-positive, AQP4-IgG-positive, and MS-related demyelinating events [8]. Delaying immunotherapy while awaiting antibody results may increase the risk of irreversible neurologic injury, including permanent vision loss [8]. Furthermore, IVIG and corticosteroids are commonly used in pediatric autoimmune encephalitis and demyelinating syndromes once infectious etiologies have been excluded [7]. The patient’s rapid clinical improvement within hours of IVIG and steroid administration further supported an immune-mediated process and validated the decision to initiate early treatment.

While pediatric MOGAD generally carries a favorable prognosis with early treatment, relapse of neurologic symptoms is common [9]. Brainstem involvement, a less common manifestation observed in this patient, has been associated with a more aggressive disease course [9]. For this reason, long-term immunotherapy with monoclonal antibodies has been suggested in selected patients. However, long-term immunotherapy does not appear to prevent brainstem attack in some patients, and long-term prognosis differs widely, with no clear associated prognostic factors [9].

## Conclusions

In this case report, we present a six-year-old male patient with MOGAD who had uncommon brainstem involvement. The patient presented with progressive neurologic symptoms and multifocal MRI abnormalities, which made initial diagnosis difficult and raised concern for several pediatric demyelinating disorders before antibody results were available. As antibody testing was delayed and there was concern for ongoing neurologic injury, treatment was started based on clinical presentation and imaging findings. The patient was given high-dose intravenous corticosteroids and intravenous immunoglobulin, which led to rapid clinical improvement. This supports an immune-mediated process and shows the importance of early treatment in suspected MOGAD.

Although pediatric MOGAD often has good outcomes with early treatment, relapse of neurologic symptoms is common, especially in patients with brainstem involvement. This case highlights the need for early recognition and initiation of immunomodulatory therapy. Future research is needed to improve long-term management and identify prognostic predictors, including treatment-related or disease-related factors.
